# Robert K. Crane—Na^+^-glucose cotransporter to cure?

**DOI:** 10.3389/fphys.2013.00053

**Published:** 2013-03-22

**Authors:** Kirk L. Hamilton

**Affiliations:** Department of Physiology, Otago School of Medical Sciences, University of OtagoDunedin, New Zealand

**Keywords:** sodium-glucose cotransporter, SGLT1, diarrhea, cholera, oral rehydration therapy

## Abstract

Dr. Robert K. Crane made major contributions to our understanding of carbohydrate metabolism and transport of the intestine over a very long and productive career. This Perspective examines, briefly, his early life and academic positions, but more importantly, this Perspective highlights his contributions to the understanding of coupled Na^+^-glucose absorption by the small intestine. I discuss how his early hypothesis of a “cotransport” of sodium and glucose ushered in and provided the physiological explanation for the clinical treatment of acute diarrhea and cholera when using oral rehydration therapy (ORT). ORT saves millions of lives each year. Certainly, humankind is better off because of Crane's hypothesis of the Na^+^-glucose cotransporter that he put forth over 50 years ago?

## Overview

I wish to start this Perspective with a few key questions. First, how does one measure the impact of a hypothesis on humankind? Secondly, how can one measure the impact of a simple idea which through its contribution to medical science, “saves” millions of lives each year? This all began with Crane's theoretical calculations for how sodium and glucose were transported across the cellular membranes of intestinal epithelial cells ~10 years before the structure of the cell membrane had been described (Singer and Nicolson, 1972)!

The focus of this Perspective is to briefly highlight the life and scientific contributions of Robert Kellogg Crane on the field of carbohydrate transport in intestinal function and implications of his work in the clinical treatment of acute diarrhea.

## The man and his scientific path

Robert Kellogg Crane (1919–2010, Figure 1A) was born in Palmyra, New Jersey to Wilbur Fiske Crane, Jr., and Mary Elizabeth McHale Crane. His father was a highly regarded farm building architect and engineer (Crane, 2010). Crane went to public schools in Palmya and eventually graduated from St. Andrew's School in Middletown, Delaware in 1938 (Crane, 2010). Afterward, Crane entered Washington College in Chestertown, Maryland. He graduated in 1942 with a B.S. in chemistry and a double minor in biology and physics. Interestingly, Crane was not all that excited about science when he entered college. However, during his freshman year he had a change of heart, which he himself attributed to lectures given by Kenneth Buxton which he described as “compelling and complete” (Crane, 2010). After a short time in the Navy, Crane began graduate school at Harvard Medical School in the medical science program. While at Boston, Crane studied biochemistry first with Eric Ball and then with Fritz Lipmann (the latter in 1953 shared the Nobel Prize in Physiology or Medicine for his discovery of co-enzyme A and its importance for intermediary metabolism with Hans Krebs, who discovered the citric acid cycle). Crane was awarded his Ph.D. in 1950 (Crane, 2010) where upon he moved to Carl Cori's Department of Biological Chemistry at Washington University School of Medicine in St. Louis. For the next 12 years whilst at Washington University, Crane continued his research in carbohydrate metabolism (Crane, 2010). Prior to Crane's arrival, in 1947, Cori and his wife Gerty Cori, shared the Nobel Prize in Physiology or Medicine for their discovery of the course of the catalytic conversion of glycogen with Bernardo Houssay, jointly awarded for his discovery of the part played by anterior pituitary lobe hormone in the metabolism of sugar. Earl Sutherland (awarded in 1971 the Nobel Prize in Physiology or Medicine for his discoveries concerning the mechanisms of the action of hormones; and instrumental in the discovery of cAMP) and Christian de Duve (who in 1974 shared the Nobel Prize in Physiology or Medicine with George Palade and Albert Claude for their discoveries concerning the structural and functional organization of the cell) were also working in Cori's Department, with de Duve closely working with the Coris' as a visiting Rockefellar Foundation fellow (Christie and Tansey, 2000). After leaving St. Louis, Crane was recruited as the Chairman of Biochemistry at the University of Chicago, and after 4 years (1966), he moved to be the Chairman of Physiology at the new Rutgers Medical School (now known as Robert Wood Johnson Medical School) where he remained until he retired in 1986 (Crane, 2010). It was therefore not so surprising that Crane, being surrounded by such illustrious scientists, was destined for greatness. During his career, Crane published over 135 research and review papers. Today, a quick search in Google Scholar determines that Crane's h-Index is currently at 50.

**Figure 1:**
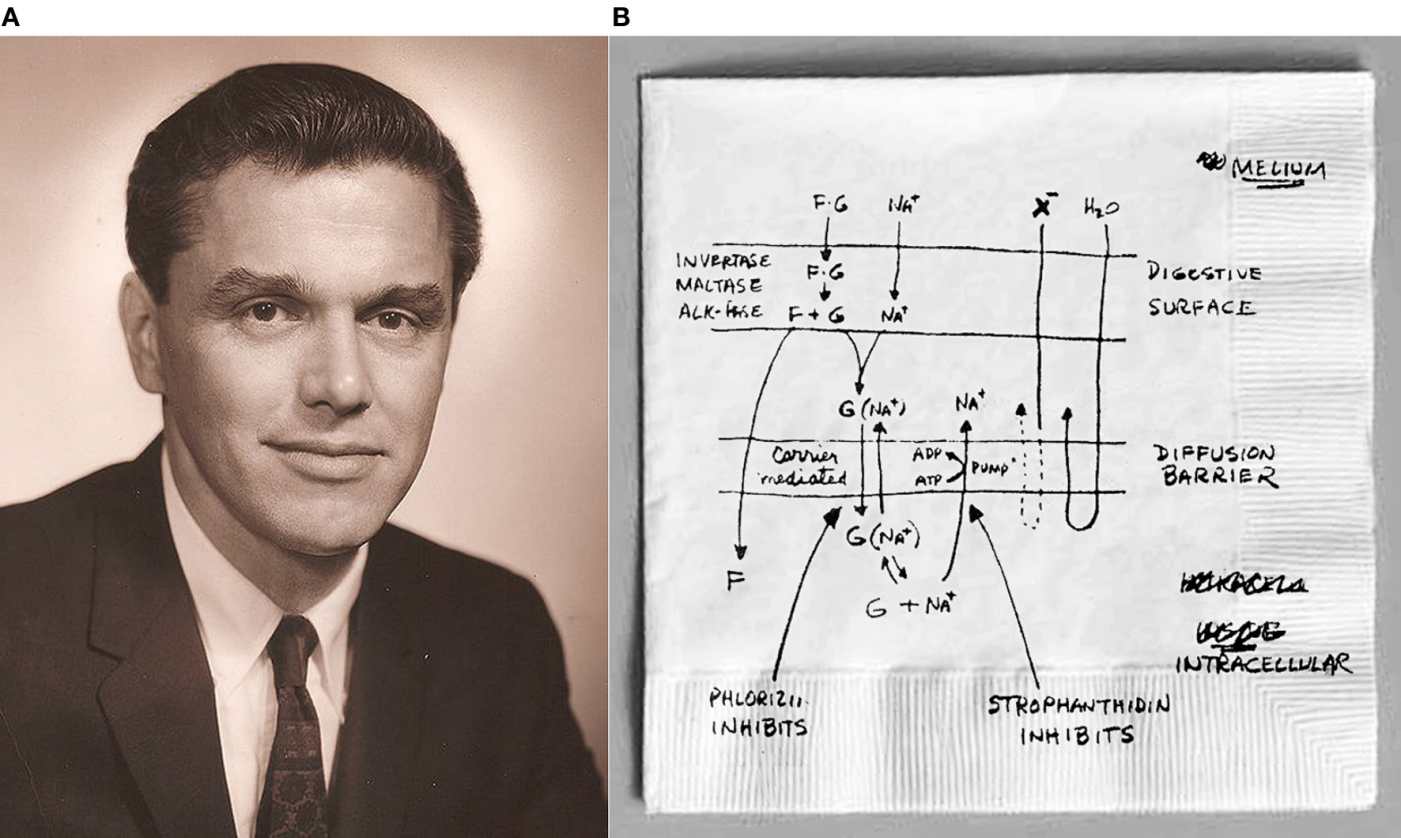
**A photo of Dr. Robert K. Crane and sketch of the Na^+^-glucose cotransport model. (A)** This is a photo of Dr. Crane in the early 1960s. **(B)** This is the model of coupled Na^+^-glucose cotransport of the small intestine. This is the hand-drawn model of the flux coupled Na^+^-glucose cotransport which Crane sketched prior to delivering his talk at the Symposium on Membrane Transport and Metabolism. It was this model that was first shown at the meetings in Prague in 1960. (Photo of Crane used with permission the Creative Commons Attribution 3.0 United States (http://creativecommons.org/licenses/by/3.0/us/deed.en) license) (Photo of the Na^+^-glucose cotransport model from “For Living History” from the American Physiological Society website http://the-aps.org/mm/Membership/Living-History/Crane).

Additional details of Dr. Crane's college and work life up to the early 1960s can be found in Crane (1983). Interested readers are also directed to the following American Physiological Society website http://the-aps.org/mm/Membership/Living-History/Crane to view an interview with Dr. Crane and additional information about him (Living History, 2010).

## Finding the Na^+^-glucose cotransporter

In the early part of Crane's career, he focused his research on phosphate, CO_2_ fixation, hexokinase, glucose-6-phosphatase biochemistry among other topics before he shifted into carbohydrate biochemistry and transport (Crane, 1960). Nonetheless, Crane was the first to champion the concept of the “cotransport hypothesis” in coupling of glucose absorption with Na^+^ transport for epithelial cells of the small intestine at international meetings in Prague in 1960 (Crane, 1960, 2010; Crane et al., 1961). As Crane described at that symposium on “Membrane Transport and Metabolism” (Crane et al., 1961) and also found in the Living History of Physiology at the American Physiological Society webpage (given above); Crane stated:

“On August 24, 1960 in a lecture presented at Prague during a Symposium on Membrane Transport and Metabolism I proposed for the first time anywhere that the fluxes of an ion and a substrate could be coupled by combining with the same reversible transport carrier in the cell membrane. In the intestinal epithelial cells that I was studying the ion was sodium and the substrate was glucose. Because of the coupling, glucose accumulation to high levels in the cells, i.e., active transport, was seen to be powered by the ATP-driven efflux of sodium ions elsewhere.”

According to Crane (2010), the final thoughts of his model were sketched quickly just prior to those meetings on what appears to be a napkin (Figure 1B). It was this sketch that he presented to the attendees of the symposium. Others in attendance at the symposium were Peter Mitchell (awarded the 1978 Nobel Prize in Chemistry for his contribution to the understanding of biological energy transfer through the formulation of the chemiosmotic theory) and Jens Christian Skou who shared the 1997 Nobel Prize in Chemistry for his work for the first discovery of an ion-transporting enzyme, Na^+^, K^+^ -ATPase, along with Paul Boyer and John Walker for their elucidation of the enzymatic mechanism underlying the synthesis of adenosine triphosphate (ATP) (Christie and Tansey, 2000).

During the 1940s and 1950s, numerous research groups on both sides of the Atlantic Ocean were working on carbohydrate biochemistry, metabolism and transport including the transport of glucose. These groups included David Fisher, Dennis Parson and David Smyth to name a few whom were working at Sheffield and Oxford and other institutions while Crane, Thomas Wilson (Harvard University) and others were working in the States. Many substantial advances in our understanding of carbohydrate biochemistry were made during this period. Interestingly, there was a Witness Seminar held in 1999 at the Wellcome Institute for the History of Medicine chaired by Professor Lord Leslie Turnberg with notable players in the field of intestinal absorption being present, including Richard Naftalin, Roy Levin, J. Ramsey Bronk, Hermon Dowling and others. At that meeting, Richard Boyd posed the question “Why wasn't sodium-coupled glucose transport discovered in the UK and, more specifically, why wasn't it discovered either in Sheffield or in Oxford?” He went on to state “… Of course, the seminal work that led to our key modern view of how sodium-coupled glucose transport occurs was a model drawn by R. K. Crane and colleagues ….” (The transcript of this meeting can be found in Christie and Tansey, 2000.)

As one reads the transcript of the 1999 meetings (Christie and Tansey, 2000), it was surprising that Crane, though retired, was not listed as a participant nor was he listed as one who sent apologies for not attending. Suffice to say, even in Crane's absence, it was duly noted and his work was rightly described as “seminal”!

Even though Crane proposed the Na^+^-glucose cotransport hypothesis in 1960, 27 years were to elapse before in 1987 Wright and colleagues cloned the protein predicted as the Na^+^-glucose cotransporter, and later referred to as SGLT1 (Hediger et al., 1987). Recently, Wright et al. (Faham et al., 2008) identified the crystal structure of a related Na^+^-galactose cotransporter (vSGLT) isolated from *Vibrio parahaemolyticus*, further validating Crane's original predictions. The reader is directed for further information concerning SGLT-1 and other human Na^+^-glucose cotransporters to a recent comprehensive review by Wright et al. (2011).

## The cellular model of Na^+^-glucose absorption

**Figure 2:**
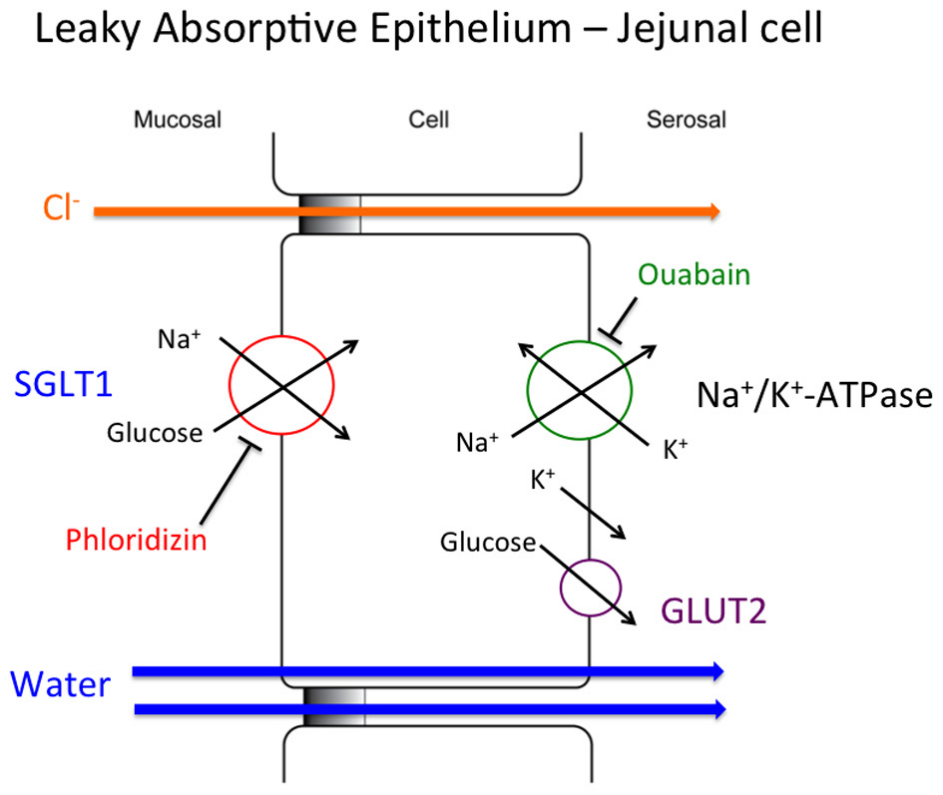
**Cellular model of the coupled Na^+^-glucose cotransporter.** The details of this cellular model are given in the text.

The now “classical” model of Na^+^ and glucose absorption (Na^+^-glucose co-transport) by the epithelial cells of the small intestine is depicted in Figure 2. The entry step of glucose across the luminal membrane is via the Na^+^-glucose cotransporter (SGLT1, encoded by the *SLC5A1* gene) and it is inhibited by phloridzin (Ehrenkranz et al., 2005) (Figure 2). The driving force for this transport protein is the Na^+^ electrochemical gradient that is generated and maintained by the basolateral Na^+^/K^+^-ATPase [Na^+^ pump; (Skou, 1957)] which is blocked by ouabain (Glynn, 1964). Na^+^ transported into the cell exits across the basolateral cell membrane via the weakly electrogenic Na^+^ pump (3Na^+^ out: 2K^+^ in) with intracellular K^+^ ions being recycled back across the basolateral membrane via K^+^ channels. This mechanism aids in the maintenance of the epithelial cell negative cellular membrane potential facilitating Na^+^ entry across the luminal membrane (Figure 2). The glucose transported by SGLT1 into the cell is either metabolized or exits across the basolateral membrane via facilitative glucose concentration gradient transport via glucose transporter 2 (GLUT2, encoded by the *SLC2A2* gene) (Augustin, 2010). The net movement of Na^+^ and glucose across the intestinal epithelium contributes to a charge separation and a small osmotic gradient which provides the driving forces for both Cl^−^ absorption via the paracellular pathway, and water absorption via both transcellular and paracellular pathways, separately (Figure 2). This physiological process results in the overall transport of Na^+^, glucose, Cl^−^, and water; four key substances needed to reduce the symptoms of acute diarrhea and cholera.

## Clinical implications—the Na^+^-glucose co-transporter and oral rehydration therapy

Infectious diarrheal diseases including cholera have contributed to hundreds of millions of deaths over the past few centuries, of which, many are children under the age of five (Synder and Merson, 1982; Taylor and Greenough, 1989). It was estimated in 1982 that there were more than 1 billion cases of acute diarrhea world-wide with nearly five million deaths alone, in the younger population (Synder and Merson, 1982). Ten years later, Bern et al. (1992) reported that the number of cases of diarrhea was maintained at one billion, however, the number of under 5-year old deaths was reduced to three million. In 2003, it was reported that deaths for this age group had dropped to 2.5 million per year (data from 1992–2000; Kosek et al., 2003). In closer examination for cholera alone, there have been eight pandemics of cholera during the past 200 years (Guerrant et al., 2003). In July 2012, the World Health Organization (WHO) estimated that there are 3–5 million cases of cholera each year with only 100,000–120,000 deaths (WHO, 2012). What has caused the incredible reduction in the death rate for individuals contracting diarrhea? The “magic bullet” has been the world wide employment of oral rehydration therapy (ORT).

There is great controversy as to whom made the greatest contribution to the discovery and use of ORT in part because early therapies which were effective, were not based on scientific discovery but, rather on folklore and natural remedies. Such remedies which included the ingestion of a mixture of molasses and rock salt, maize, yams, rice water, coconut juice, green bananas (among other natural products) have been recorded during the past 3000 years and used to reduce the loss of water from the body (Rao, 2004) and therefore alleviating the symptoms of diarrhea.

While ORT stumbled around in the dark for many years there were a number of scientific “kingpins” in the nineteenth century who actively contributed to our scientific knowledge. Of these, O'Shaughnessy (1831) was one of the early pioneers who attempted to not only treat but understand the scientific basis of watery diarrheas, observing that patients with such ailments as exhibiting excessive loss of salt and water in the stool. He posited that the dehydration and changes in blood chemistry of patients with cholera, which mimicked those seen for yellow fever, could be better treated by giving patients intravenous salt and water as a remedy. He was one of the first to try and explain why the ingestion of weak alkali salts with or without starches in tepid water, was effective. A phenomenon he attributed to a cleansing of the blood. Early investigations into the infectious disease basis for cholera were made a short time later by Dr. Jack Snow during the 1850s, who first attributed the spread of cholera to a problem with the drinking water supply by plotting patient deaths with distances from drinking water wells in London (Snow, 1855; Schultz, 2007). While the later findings certainly advanced our understanding of the need for water cleanliness and sanitation it was not until the 1940s, that D. C. Darrow (a pediatrician) refocused the attention of many within the scientific field on the fact that understanding the exact changes in the ionic composition of body fluids in patients with infantile diarrhea would be paramount for the successful treatment of the disease (Darrow et al., 1949). Captain Robert Phillips led the fight against the 1961 pandemic of cholera in the Philippines (Phillips, 1963). He used a solution that was composed of a low sodium electrolyte that was “isosmolar” and that he used glucose as a non-electrolyte to replace the reduced sodium (Ruxin, 1994), therefore, some credit Phillips to be the first individual to use an ORT-type approach. He was awarded the Albert Lasker Medical Research Award in 1967, the United States' preeminent medical award, for “… his contributions to the understanding of the mechanism of death in cholera, and the development of a life-saving method of treating it” (Phillips, 1967; Savarino, 2002). During the 1950s many biochemists and physiologists were studying the properties of carbohydrate transport of the mammalian intestine. Fisher and Parsons (1950, 1953) examined transport properties of glucose across the rat small intestine in early 1950s. Riklis and Quastel (1958), some say, lead the way in understanding the importance of sodium in the active transport of glucose by the guinea pig intestine (Ruxin, 1994). While, work by Wilson and Crane (1958) and Crane and Krane (1959) helped explain the mechanism of active transport of glucose and the need for the presence of sodium. Finally, Schultz and Zalusky (1964a,b), using Ussing chambers and the short-circuit current technique, examined the Na^+^ fluxes, short-circuit current, transmural potential difference and glucose transport of the isolated rabbit ileum. All of these individuals, and many more who have not been highlighted here, have made major contributions to our understanding of Na^+^ and glucose transport across the intestinal epithelium and their work laid the firm foundation for ORT.

## Final thoughts

So, how does Crane's contribution of the Na^+^-glucose cotransporter “fit” in with the development of ORT? Well, this is actually a complicated question. There is no doubt that Crane's model is the basis of the cellular mechanism of Na^+^-glucose absorption by the small intestine. Indeed, the Na^+^-glucose cotransporter is the “heart” of the cellular model and of the successful use of ORT. Without the Na^+^-glucose cotransporter there would be no ORT, and thus, there would of been hundreds of millions of people who would not have led a full life. So, I am drawn back to the words of Richard Boyd who described Crane's work as “seminal.”

It is obvious that Crane made major contributions to the field of carbohydrate metabolism and transport. I will end this Perspective with a quote that has been repeated in science literature and in the popular press “… the discovery that sodium transport and glucose transport are coupled in the small intestine, so that glucose accelerates absorption of solute and water, was potentially the most important medical advance this century [20th century]… (The Lancet, 1978).” Certainly, this is an appropriate testimony to the memory of Robert Kellogh Crane.

### Conflict of interest statement

The author declares that the research was conducted in the absence of any commercial or financial relationships that could be construed as a potential conflict of interest.
